# Accurate spectra for high energy ions by advanced time-of-flight diamond-detector schemes in experiments with high energy and intensity lasers

**DOI:** 10.1038/s41598-021-82655-w

**Published:** 2021-02-04

**Authors:** Martina Salvadori, F. Consoli, C. Verona, M. Cipriani, M. P. Anania, P. L. Andreoli, P. Antici, F. Bisesto, G. Costa, G. Cristofari, R. De Angelis, G. Di Giorgio, M. Ferrario, M. Galletti, D. Giulietti, M. Migliorati, R. Pompili, A. Zigler

**Affiliations:** 1 Università d Roma La Sapienza, Piazzale Aldo Moro 5, Rome, Italy; 2 INRS-EMT, Varennes, Québec Canada; 3 ENEA Fusion and Technologies for Nuclear Safety Department, C.R. Frascati, Via Enrico Fermi 45, Frascati, Rome, Italy; 4 Industrial Engineering Department, University of Rome “Tor Vergata”, Rome, Italy; 5 INFN-LNF, Frascati, Rome, Italy; 6 Department of Physics, University of Pisa, Largo Bruno Pontecorvo 3, Pisa, Italy; 7 GoLP Instituto de Plasma e Fusão Nuclear, Istituto Superior Técnico, Universidade de Lisboa, Lisbon, Portugal; 8 INFN of Pisa, Largo Bruno Pontecorvo 3, Pisa, Italy; 9 INFN Sezione di Roma, Piazzale Aldo Moro 2, Rome, Italy; 10 Racah Institute of Physics, Hebrew University, Jerusalem, Israel

**Keywords:** Physics, Laser-produced plasmas, Characterization and analytical techniques

## Abstract

Time-Of-Flight (TOF) methods are very effective to detect particles accelerated in laser-plasma interactions, but they show significant limitations when used in experiments with high energy and intensity lasers, where both high-energy ions and remarkable levels of ElectroMagnetic Pulses (EMPs) in the radiofrequency-microwave range are generated. Here we describe a novel advanced diagnostic method for the characterization of protons accelerated by intense matter interactions with high-energy and high-intensity ultra-short laser pulses up to the femtosecond and even future attosecond range. The method employs a stacked diamond detector structure and the TOF technique, featuring high sensitivity, high resolution, high radiation hardness and high signal-to-noise ratio in environments heavily affected by remarkable EMP fields. A detailed study on the use, the optimization and the properties of a single module of the stack is here described for an experiment where a fast diamond detector is employed in an highly EMP-polluted environment. Accurate calibrated spectra of accelerated protons are presented from an experiment with the femtosecond Flame laser (beyond 100 TW power and ~ 10^19^ W/cm^2^ intensity) interacting with thin foil targets. The results can be readily applied to the case of complex stack configurations and to more general experimental conditions.

## Introduction

Thanks to the development of new high energy and high-power lasers^1^, during the last decades the field of laser-plasma particle acceleration has experienced a significant evolution and expansion^2–4^. The interest in this field arises from the possibility to produce, with a compact system, intense bunches of high energy charged particles that can be used for several notable applications^5,6^, including inertial confinement fusion^7^. According to the laser parameters, different acceleration processes can take place during the laser-matter interaction^2–4^. In the Target Normal Sheath Acceleration (TNSA) a high-power laser (up to the PW power and to the ${10}^{21}\mathrm{ W}/{\mathrm{cm}}^{2}$ intensity) is focused on a solid target, leading to hot electrons generation at the front side. These electrons can penetrate the target, reach its rear side and escape from it. A strong sheath field is created due to charge separation on the rear side of the target. Protons and heavier ions are thus accelerated by the remarkable electric fields and follow the electron expansion in the vacuum chamber^2–4^.

The study of the acceleration processes that take place in the frame of the laser–plasma interaction requires the development of dedicated diagnostics able to characterize the properties of the accelerated particle beams and thus to outline the differences among the different possible acceleration mechanisms. These diagnostics have to meet some important requirements depending on the characteristics of the accelerated particle beams. Among these, high sensitivity and high energy resolution are needed for the accurate spectrum reconstruction of the accelerated ions. Moreover, the notable dependence of these beams on the angle of emission requires a precise characterization of the particle angular distribution at different directions. This can be done by methods employing stacks of passive detectors as for example Radiochromic films (RCF)^8,9^, which allow to reconstruct the beam divergence providing high spatial resolution (up to ~ 2.5 mμ) and sensitivity, but have an intrinsic low energy resolution (~ 1 MeV) and do not distinguish among different ion species. Imaging Plates (IP)^10^ and CR39^11^ are also commonly adopted for ion detection. The first may have high sensitivity and dynamic range of 16 bit, with spatial resolution normally limited to 25–50 mμ by the type of IP and by the scanner used for retrieving the impressed image. CR39 is a track detector, and has extremely high sensitivity, allowing for single particle detection and resolution commonly limited to a few microns, at most. In some configurations, from the track dimension it is possible to retrieve some information on the energy of the incoming particle, even if with low resolution of the MeV order. The three mentioned detectors are commonly adopted when performing time-integrated measurement since they do not allow to have any temporal discrimination. Their spatial resolutions allow them to give accurate representation of the angular particle emission properties, and in some cases information on the number of detected particles can be achieved also with good accuracy, for example with the CR39. They are also immune to the intense Electromagnetic pulses (EMPs) in the radiofrequency-microwave range generated in laser-matter interactions of high energy and intensity^12–18^. On the other hand, they cannot intrinsically supply accurate information on particle velocity and energy.

Electrostatic-magnetostatic spectrometers of the Thomson type^19,20^ are instead able to recognize the different charge-over-mass ratios for the incoming ion species and to reconstruct their spectra, but do not have any angular discrimination. The physical dimension of these devices usually gives notable difficulties to place several of them at different directions, on the purpose to retrieve information on the angular beam emission, especially in the cases where the vacuum chamber is of limited dimension.

An additional requirement that the ideal diagnostic system has to satisfy is the capability to work at high repetition rates (i.e. up to kHz), so without having to open the experimental chamber after each shot. This task cannot be fulfilled when passive detectors such as RCF, CR39 or Imaging Plate are used, both in the stack configuration or within Thomson spectrometers. Indeed, the significant limitation of these detectors is that they cannot be used for online detection, being the process of information retrieving not immediate and in the case of CR39 definitely cumbersome. In the case of Thomson spectrometers, active expensive solutions, such the coupling of a phosphorous to a microchannel plate (MCP), can be used^20^. The MCP response is of the order of a few ns, i.e. the fluorescence time, allowing to work in a continuous or gated biasing. In some conditions these can be used for real-time particle detection. The MCP typical spatial resolution, when used for imaging or coupled to electric–magnetic spectrometers, is limited to 4–25 µm by the diameter of the MCP capillaries, although is usually worst due to the common set-ups including pin-holes and drift space. Thanks to the electron multiplication that can span over several decades, they can have very high sensitivity and dynamic range. On the other hand, this leads to a low signal-to-noise ratio with respect to other solutions. Moreover the high cost of such devices limits the number of possible detectors to be fielded, and their use can be in some cases highly problematic due to the high values of EMP fields typical of high energy and high intensity laser–plasma interactions^12–18^, that can highly affect the operation of the related electronics. The employment of scintillators together with differential filtering technique has been also proposed^21^ to retrieve the proton beam profiling as well as a coarse energy estimation while working at high-repetition rate.

Eventually, the Time-of-Flight (TOF) technique^9,22–26^ is commonly adopted to obtain time-resolved characterization of the accelerated ions, overcoming most of the limitations met by the detection methods previously described. It has indeed the potential to meet all the discussed requirements. The possibility to work with real-time readout systems, without the need of opening the chamber after each shot, makes the TOF an ideal candidate for ion diagnostics in high repetition-rate experiments. Energetic ions are usually detected using semiconductor devices made of SiC or diamond^27–29^, or scintillators^26,30,31^. In some cases systems composed by MCP coupled to phosphorous may be also used.

As Thomson spectrometry, TOF technique is not able to give information on the angular distribution of the accelerated particles. Nevertheless, in general the higher compactness, the reduced dimension and cost of some type of TOF detectors, coupled to optimized real-time data retrieving—no matter how many of them are fielded—allow to install several of them along different lines of observation, even at small relative distances from the target and at rather similar angles, providing a simultaneous high-resolution real-time and high-sensitivity measurement of the particle energy distribution along different directions. Thus, all these features allow to effectively overcome the problems encountered when trying to fulfil the same task by placing several Thomson spectrometers around the chamber, and that normally highly limit the number of detection directions to a few units^32,33^.

Nevertheless, the TOF method has historically shown significant limitations when applied to experiments with high energy and intensity lasers, where both high-energy ions^34,35^ and remarkable levels of EMPs are generated. Protons up to several tens of MeV are here produced^36^, with typical spectra decreasing with energy. So, both high sensitivity and high resolution are required for a suitable description of the most energetic part of the ion emission. Chemical Vapor Deposition (CVD) diamond detectors have the potential to satisfy both these needs, but with the TOF technique energy resolution and sensitivity have somehow opposite requirements. Indeed, improved energy resolution is here commonly obtained in two ways.

1. By using fast detectors. In TOF, energy resolution is linked to time resolution, and the detector response velocity is mainly limited by the thickness of the active sensitive region, associated with the time of charge collection to the electrodes. The typical monocrystalline diamond detector response to a single 5.486 MeV α particle emitted by ^241^Am decay can have FWHM up to 8 ns for a detector with 500 µm thickness^37^, up to 1.1 ns for one with just 100 µm thickness^37^ and up to 0.8 ns or below for one with 50 µm thickness^29,38^. So thinner detectors should be preferred for better energy resolution, but on the other hand energetic protons will pass through them more easily, leaving there only a small amount of their energy, and this will lead to reduced sensitivity of the detector to them. It was shown by Monte-Carlo SRIM code simulations^39^ that protons with energies up to E_THRS-100_ ~ 4.6 meV will be stopped within the active region of the 100 µm diamond, releasing all their energy in the detector. Alternatively, those with much higher energy will lose a very limited portion of it. If the signal produced in the detector is small with respect to the background noise, the information on the associated particles can be lost. As observed, the emitted proton spectrum in these experiments decreases with energy, and thus this problem is even more significant for the most-energetic protons. For particles passing through the detector whose produced signal is still reasonably higher than the background, it is possible to reconstruct, by means of suitable numerical simulations^24^, the initial particle energy from the small portion released in the detector and thus estimate the associated number of detected particles. But this will commonly suffer of low precision due to the related large confidence interval. So, high energy resolution will be achieved but with low resolution for the number of particles in the spectrum.
2. By placing the detectors at larger distances from the target. However, this reduces the overall sensitivity by decreasing the solid angle of detection and thus the number of particles intercepted by the detector.

For these reasons, whenever improved sensitivity for the most energetic ions is required, thicker detectors are used^24,37,40^, but at expenses of the time resolution^37^, and thus of the related energy resolution accordingly. One key issue for the sensitivity threshold is the background noise associated with the technique. This is due to the intrinsic noise of all the electronic equipment used, and in particular of the oscilloscope, and to the external electromagnetic noise coupled to the overall TOF detecting chain. It is well known that in energetic high power laser facilities the intense laser-matter interaction generates strong EMPs in the radiofrequency-microwave range^12^, which remarkably affect the signal-to-noise ratio of the electronic devices placed inside and nearby the vacuum chamber, and that commonly scale with the laser intensities^18,41^. Since TOF measurements require time-resolved detectors connected with fast electronic readout systems, the presence of remarkable levels of EMPs has, so far, strongly hindered the employment of this technique in experiments with intense ultra-short pulses at very high-power regimes, where instead their features would have been very useful.

**Figure 1:**
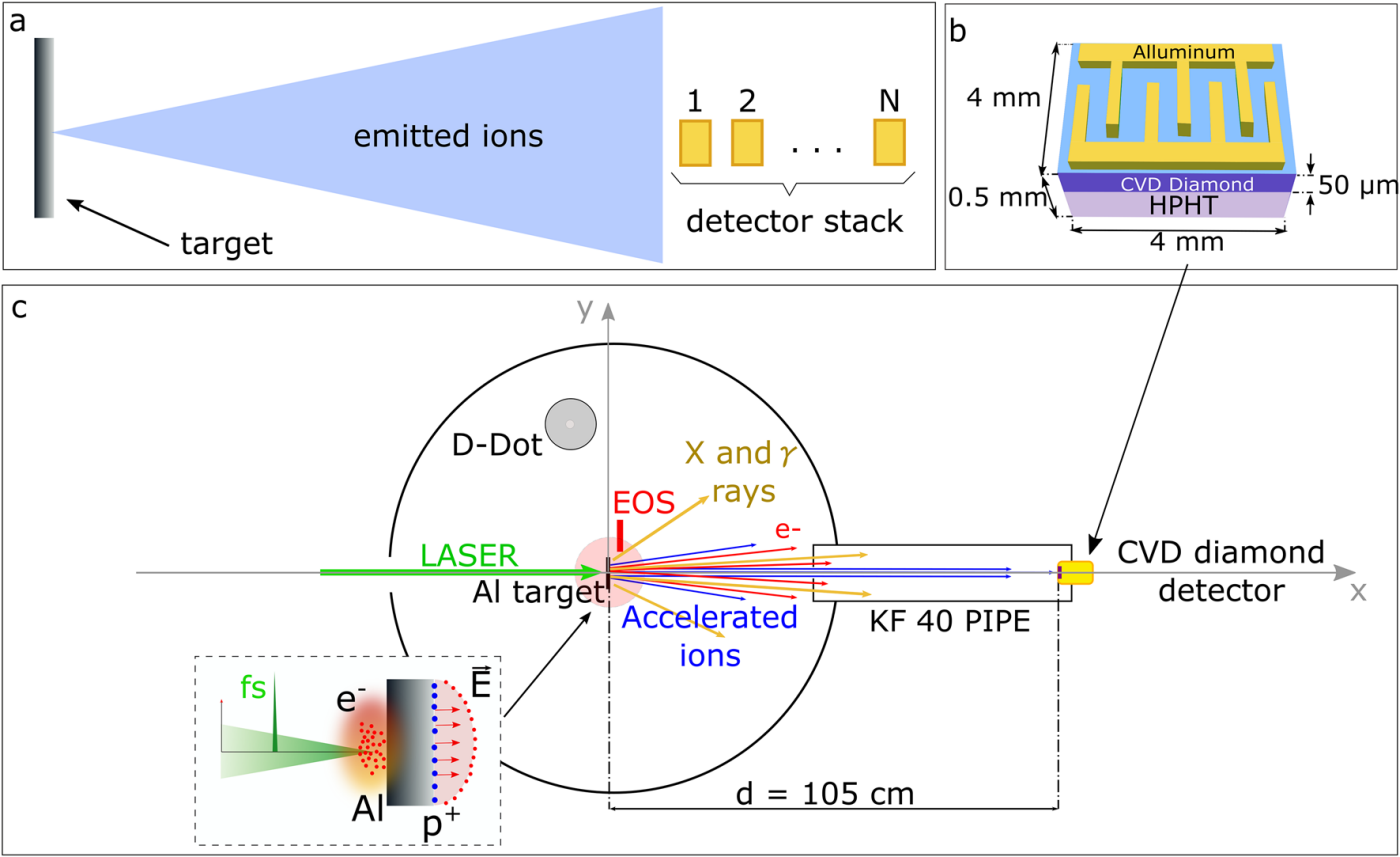
(**a**) Basic scheme of the advanced TOF detector. (**b**) Schematic representation of the diamond detector in planar-interdigital configuration. (**c**) Experimental chamber layout; in the inset: a scheme of the laser-plasma interaction highlighting the TNSA mechanism, being E the electric field generated on the rear side of the target by the escaping hot electrons.

In order to fully exploit the TOF potentiality, we present here the basic idea to use a composite structure made of a set of several time-resolved detectors, which can be positioned as a stack, one after to the other along the direction of the impinging ions emitted from the laser-matter interaction, as shown in Fig. 1a. Filters of different materials and thicknesses can be also used in front of the detectors to condition the incoming particle beams. Here we consider diamond detectors only, but the methodology can be applied also to solid-state detectors made of other semiconductors, such as for instance SiC detectors. The thickness of each detector in the stack can be chosen to optimize the performances of the whole sensor. The total length would be given by the sum of all the detector thicknesses in the stack. In particular, the use of thin (i.e. 50–100 µm) modules could give high energy resolution and nice sensitivity to high-energy protons, at expenses of a larger number of modules in the stack. In addition, using thin diamond layers allows for a high radiation hardness of the whole diamond detector^42,43^.

The main advantage of this novel design is the capability to detect high-energy particles with good sensitivity, without sacrificing energy resolution. The sensitivity features of thick diamonds can thus be replaced with the layered multiple thin diamonds, insuring fast time response and high radiation hardness. It is worth to underline that the technology of diamond detectors is already mature for such complex structures. Devices based on multiple diamond detectors are reported in the literature for other applications: proton recoil telescope based on two diamonds for fast neutrons diagnostic^44^ and monolithic diamond-based ΔE–E charged particle telescope for identification of fragments coming from the nuclear reactions^45^.

Whenever these schemes will be applied to detect ions emitted by intense matter interactions, with high energy and intensity laser pulses up to the femtosecond and attosecond time-scales, the main limitations to the method sensitivity will be given by the coupling of giant EMPs (up to several MV/m levels^12^) to the related electronic devices. This is known to be a major issue especially for devices working at larger frequency bandwidths^12^ and will thus be more serious for the fast (i.e. thin) detectors. The capability to deal with this fundamental issue is one of the key factors for the successful and effective implementation of these methodologies in the future and will be thus here described in detail.

This work is meant as the advanced developing of a methodology capable to fully take advantage of the properties of the TOF methods applied to diamond detectors, ensuring high sensitivity, high resolution and high signal-to-noise ratio in environments heavily affected by remarkable EMP fields. For this reason we describe here a detailed study on the use, the optimization and the properties of a single module of the stack, in experiments with high energy and high intensity laser pulses with femtosecond time-duration interacting with thin foil targets, on the purpose to apply the associated considerations to the case of complex stack configurations and to more general experimental conditions.

The method has been here tested in the condition of a laser-matter interaction where the dominant acceleration mechanism is the widely studied TNSA at moderate interaction intensities. The specific and classical regime used in the present work is extensively reported in literature^2–4,46–51^. This was done on the purpose to have a well-known reference context to exploit as useful benchmark for the testing and the validation of the techniques here discussed to retrieve accurate calibrated spectra of laser-plasma accelerated protons.

## Results

### Experimental setup

The diagnostic methodology was tested and optimized during an experimental campaign performed on 10 μm aluminium flat targets, irradiated with the FLAME Ti:Sapphire laser at INFN, Laboratori Nazionali di Frascati, having 800 nm fundamental wavelength, 3 J maximum energy, 30 fs pulse duration and 25 µm focal spot, ${10}^{-9}$ contrast ratio, for 100 TW peak power and $2\times {10}^{19}$ W/cm^2^ maximum intensity on target^52^. The targets were fixed on an ad-hoc multi-target holder. During the experiment two main diagnostics were employed. An Electro-Optical Sampling (EOS) device was used to measure the longitudinal profile of the accelerated electron bunches^9,53–56^. Accelerated ions were detected by a CVD diamond detector, schematically shown in Fig. 1b, mounted at the end of a 105 cm long TOF line placed behind the target, as shown in Fig. 1c. The employed diamond was meant to be used as a single module of the stack structure previously described.

**Figure 2:**
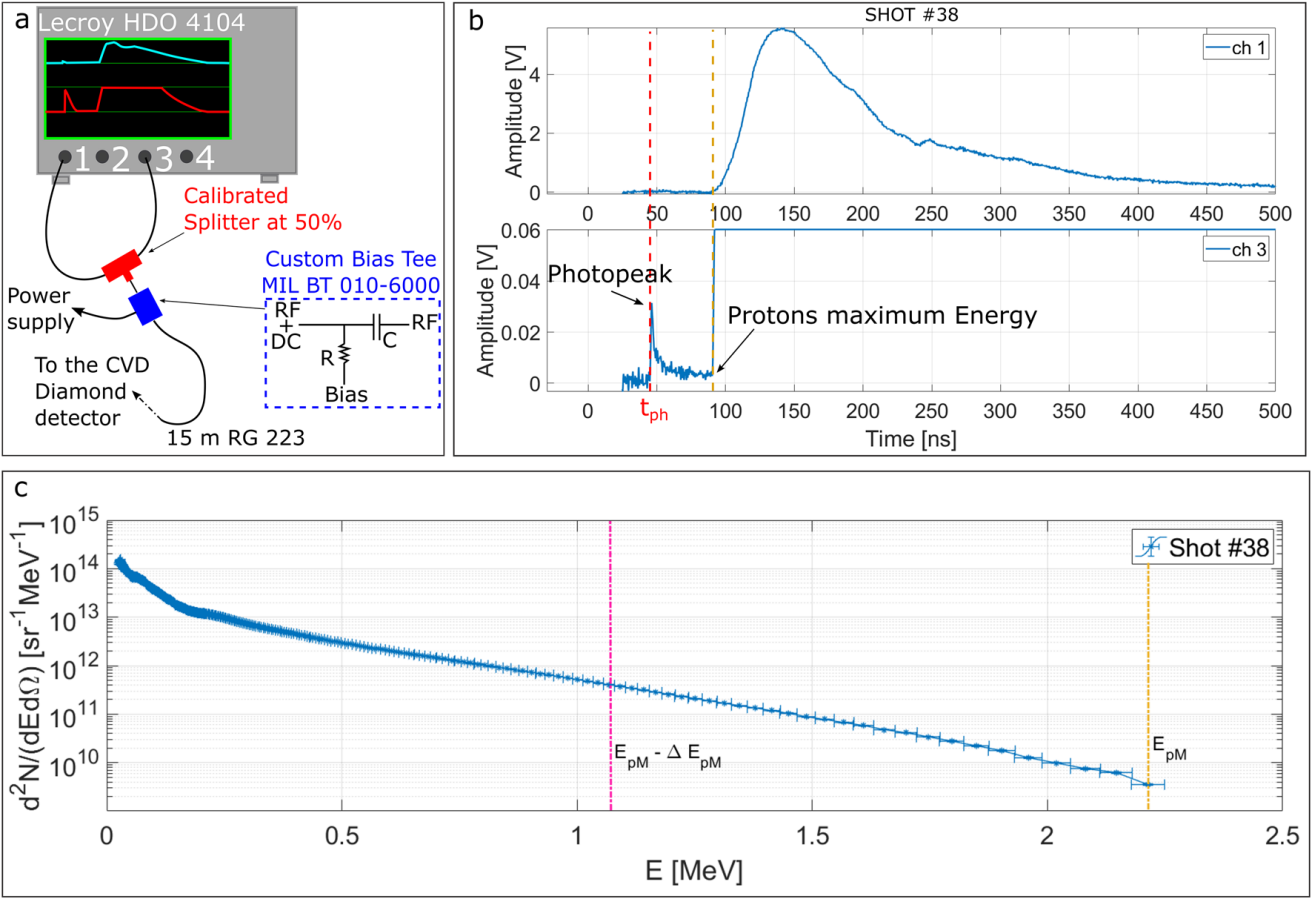
(**a**) The acquisition system configuration for the CVD diamond detectors and (**b**) a typical TOF signal acquired with the described system on channel 1 (Ch1) and channel 3 (Ch3) of the oscilloscope (**c**) proton spectrum obtained for the Shot #38 with the described procedure. The vertical yellow line indicates the maximum proton energy (${E}_{pM}$) and the magenta line corresponds to the energy where the ion contribution to the spectrum ends (${E}_{pM}-\Delta {E}_{pM}$).

The TOF measurements employing diamond detectors are intrinsically characterized by large dynamic-range signals. To retrieve all the useful information from them, to accurately estimate the energy of the incoming particles and to compute their spectrum, it is necessary to distinguish the finest details without cutting the most intense portion of the signal. The signal collected from the diamond detector was thus divided in two parts by a calibrated splitter, both having the same shape but half of the original amplitude and stored in two different channels of the same oscilloscope. The acquisition set-up is schematically depicted in Fig. 2a.

### Experimental TOF measurements without filter

A typical TOF measurement obtained with this set up is shown in Fig. 2b. It mainly consists of two parts: the *photopeak* and the *particle contribution*. The first is generated by the ionizing electromagnetic radiation emitted during the laser-matter interaction and, depending on the specific experimental conditions, can be higher or lower than the second. The photopeak is a clear and very useful signature of the laser-matter interaction instant. Its temporal position gives a reliable absolute reference for determining the arrival times-of-flight of particles from the target to the detector, as discussed in detail later. The photopeak is then followed by a tail produced by the detection of fast electrons^24,25,37^, and of UV and soft X-rays coming from the expanding plasma, whose temperature is decreasing along time. The duration of this tail can be up to several nanoseconds.

The second main peak comes instead from the time superimposition of particles arriving to the detector. Both ions and electrons can produce a signal, but the electrons arriving at the same time instants of the ions have much lower energies, and thus their contribution result typically negligible with respect to them. Anyway, whenever required, the application of a very low magnetic field close to the diamond is usually sufficient to deflect them and to avoid their arrival to the detector, practically without modifying the trajectories of the ions.

The signal reported in Fig. 2b was obtained for the shot #38, performed with $2.5$ J energy and $1.7\times {10}^{19}$ W/cm^2^ intensity. It is evident that channel one (Ch1 in the Figure) provides information about the main signal generated inside the diamond but, because of the finite dynamic range of the scope, there is poor resolution on the smaller details which, instead, can be obtained from channel three (Ch3 in the figure) which was set to a finer scale. Since the two channels of the same oscilloscope are accurately synchronized, from Ch3 the temporal position of the photopeak was retrieved and used as absolute reference for data shown in Ch1. The consistency of this comparison is confirmed by the coincident temporal position of the protons maximum energy given by the initial point of the main signal (yellow dotted line in Fig. 2b).

The calibrated proton energy spectrum obtained from the TOF signal shown in Fig. 2b is reported in Fig. 2c. This was retrieved applying the methodology described in the “Methods” section.

### Experimental TOF measurements with filter

**Figure 3:**
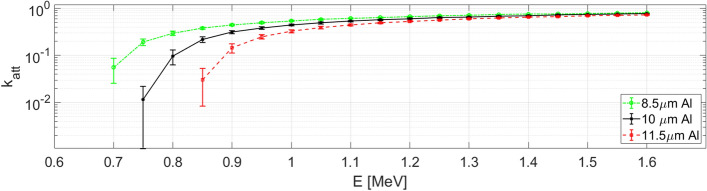
Attenuation factor *k*_*att*_ for the (10 ± 1.5) µm Aluminium filter obtained from SRIM simulations, in particular for its nominal value of 10 µm, and for the two extremes of the interval: 8.5 µm and 11.5 µm.

In some shots of the present campaign an aluminium filter of $\left(10\pm 1.5\right)$ μm thickness was placed in front of the interdigital diamond detector. Filters are usually employed to cut the contribution of heavier ions from the detected signal, with a little loss of information on protons. We define E_i-cutoff_ the maximum energy of ions of a given species fully stopped by this specific filter. According to the simulations performed with the SRIM code, for the nominal 10 µm thickness of the used filter, it was found E_p-cutoff_ = 750 keV for protons^57^ (see Fig. 3). Simulations also gave cutoff energies of 11.45 MeV for carbon, 13.7 MeV for nitrogen and 15.84 MeV for oxygen ions, the main species expected to be accelerated in this type of experiments as impurities on the target surface, and 25.2 MeV for aluminium ions coming from the bulk target. Moreover, in Fig. 3 the energy attenuation for protons able to pass through the filter was computed for the same 10 µm thickness and for the minimum and maximum values of thickness according to the filter tolerance (8.5 μm and 11.5 μm, respectively). This attenuation factor is obtained by the relation ${k}_{att}\left({\mathrm{E}}_{\mathrm{in}}\right)={E}_{out}/{E}_{in}$ where ${E}_{in}$ is the energy of the particle impinging on the filter and ${E}_{out}$ is the energy of the particle that crossed the filter.

**Figure 4:**
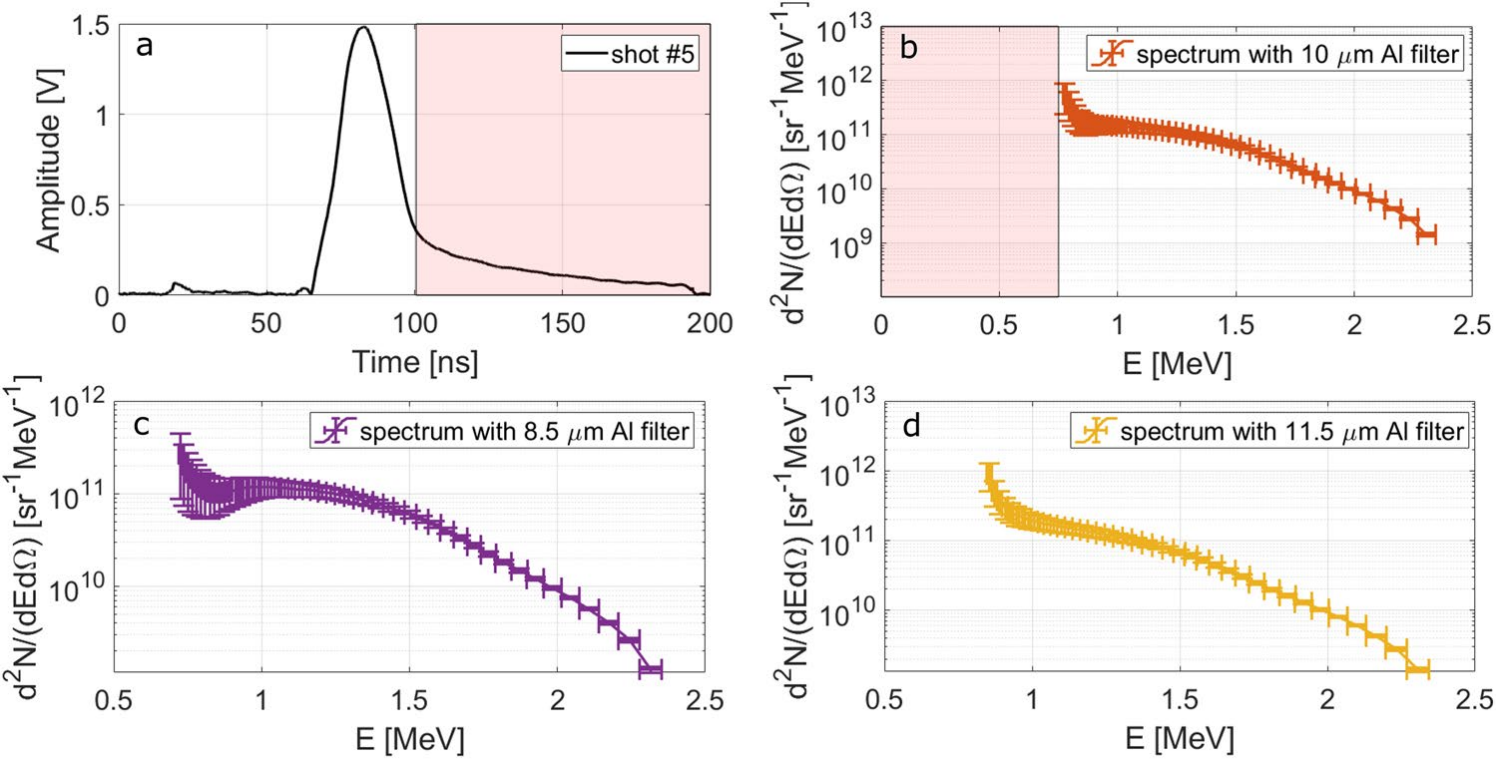
(**a**) Signal obtained for shot #5 with the Al filter covering the diamond detector. (**b**–**d**) Proton spectrum obtained for this shot by taking into account the effect of the Al filter of thickness 10 µm (**b**), 8.5 µm (**c**) and 11.5 µm (**d**).

The signal obtained for the shot #5 (E_L_ = 2.65 J, I_L_ = 1.8 × 10^19^ W/cm^2^), performed employing the aluminium filter in similar conditions to the previous #38, is reported in Fig. 4a. Figure 4b–d show the proton spectra obtained for the shot #5 using the nominal thickness of the filter and its upper and lower limit on the basis of the tolerances, i.e. 8.5 µm and 11.5 µm. The increase of the error bar on the amplitude is due to the associated tolerance in the attenuation of the aluminium filter given in Fig. 3 and it is more relevant for low energy particles. Indeed, low-energy particles are those that are more affected by the presence of the filter and, especially near to the filter cutoff energy, a small difference in its thickness can result into a quite big difference in the respective attenuation factor.

**Figure 5:**
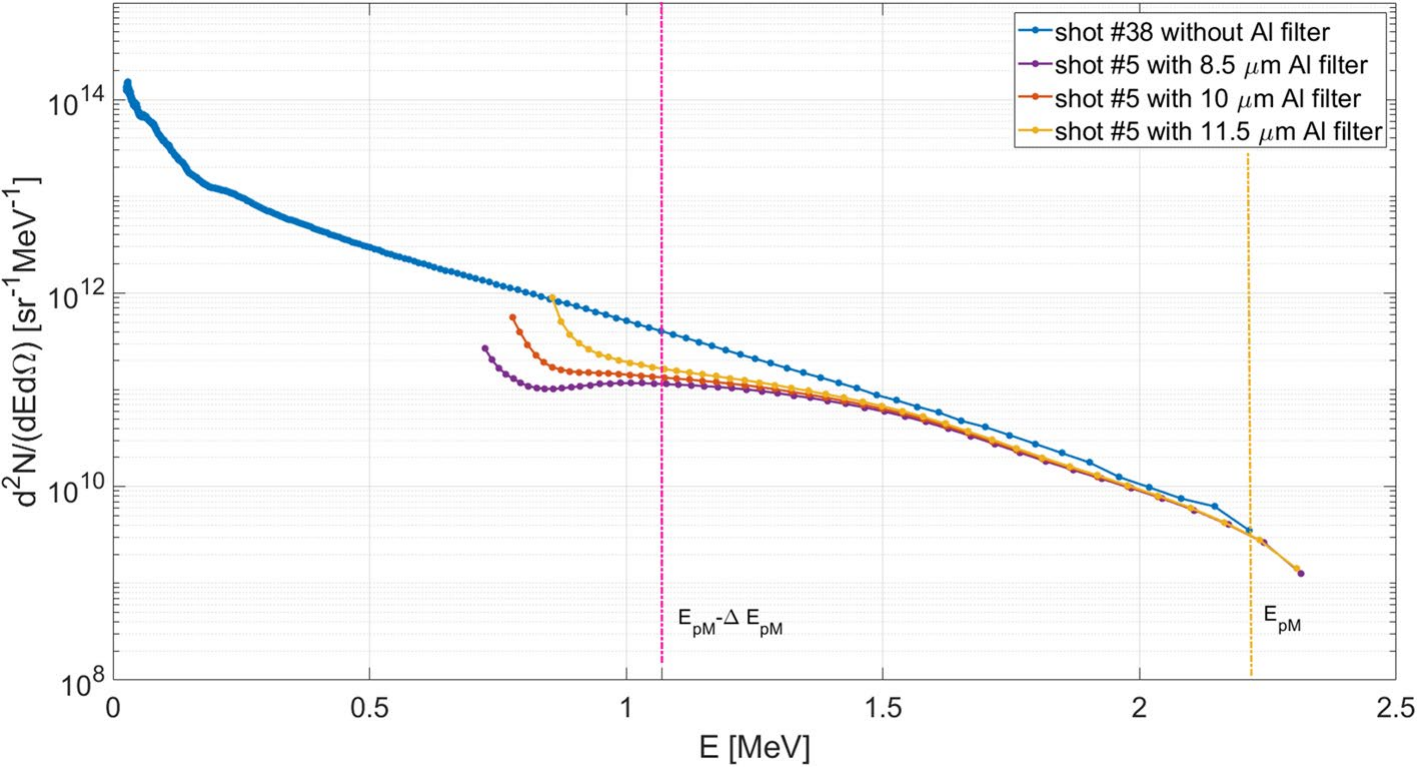
Comparison between the spectra obtained from the signals acquired for the shot #38, without the Al filter, and shot #5 with the Al filter for the three thicknesses, according to the nominal tolerances. The vertical yellow line indicates the maximum proton energy (${E}_{pM}$) obtained during shot #38 and the magenta line corresponds to the energy where the ion contribution to that spectrum ends (namely ${E}_{pM}-\Delta {E}_{pM}$), as described in the section “Estimation of the energy range for pure proton detection”.

In Fig. 5, the spectrum for shot #38 performed without any filter and the three spectra for shot #5 obtained applying the spectrum reconstruction method considering the nominal thickness of the applied filter (red curve) and its higher and lower extremes (yellow and purple curve respectively) given by the tolerances in the filter thickness measurement (already shown in Fig. 4b–d), are compared on the same graph. Here the error bars are not reported for a better visualization. It is possible to notice that the shots were rather similar in terms of the spectrum, and the use of the filter allowed to down-extend the proton energy range, at expenses of the larger errors introduced in the lower energy part of the obtained spectrum.

### Estimation of the energy range for pure proton detection

In laser-matter interaction experiments, different ion species are classically accelerated and arrive to the detector usually superimposed in time. Indeed, in a classical TNSA scheme, ions are accelerated because of the electrostatic potential developed in the charged sheath formed in the target thanks to the electron emission^2–4,41^. Because of this potential, ions are accelerated with a typical spectrum represented by a decreasing exponential function^4^. A broad energy distribution is given by the acceleration of particles from different target depths and by the inhomogeneous electron distribution in the sheath^4,41^. As a general discussion, for a test ion accelerated by the *Φ* potential drop in the sheath, having ${A}_{i}$ mass number, ${z}_{i}$ charge state and ${\mathrm{Z}}_{i}$ atomic number, it is possible to write that the energy is E_i_ = z_i_ q_e_ Φ, where q_e_ is the elementary charge^3^. For ion energies up to several tens of MeVs non-relativistic formulas can be used, and thus the ion time-of-flight at the diamond detector will be:

1 $${t}_{i}=d\sqrt{\frac{{A}_{i }{m}_{p}}{2{z}_{i}{q}_{e}}}={t}_{p}\sqrt{\frac{{A}_{i }}{{z}_{i}}}$$

where ${\mathrm{m}}_{\mathrm{p}}$ is the proton mass and ${t}_{p}$ the time of arrival to the same detector of a proton accelerated by the same potential drop. Since for any ion, except for protons, it is ${\mathrm{A}}_{\mathrm{i}}>{\mathrm{z}}_{\mathrm{i}}$, then it will always be ${\mathrm{t}}_{\mathrm{i}}>{\mathrm{t}}_{\mathrm{p}}$. Thus, protons will reach the detector much before than other ions accelerated in the same experiment. Moreover, there will be a time window where only protons can reach the detector. If we indicate with t_pM_ and t_iM_ the times of arrival to the detector of the proton with the maximum energy and of the corresponding ion with the maximum energy, generated respectively by the same acceleration process, the time window will be (t_pM_, t_iM_) = (t_pM_, t_pM_ + Δt_p_). In the limiting case of fully-stripped ions (i.e. ${\mathrm{z}}_{\mathrm{i}}={\mathrm{Z}}_{\mathrm{i}}$), it is possible to write:

2 $$\Delta {t}_{p}= {t}_{pM}\left(\sqrt{\frac{{A}_{i }}{{Z}_{i}}}- 1\right)$$

It is well known that for any element (with exception of protons), it is ${\mathrm{A}}_{\mathrm{i }}\ge 2{\mathrm{Z}}_{\mathrm{i}}$, being the equality true for light elements (C, N, O, …) classically accelerated in intense laser-matter experiments. So, it is:

3 $$\Delta {\mathrm{t}}_{\mathrm{p}} \ge {\mathrm{t}}_{\mathrm{pM}}\left(\sqrt{2 }- 1\right)$$

It is possible to determine the same condition in the energy domain. So, the proton energy range associated with the extremes of the time interval of Eq. (2) can be written as (E_iM_, E_pM_) = (E_pM_ − ΔE_p_, E_pM_), where:

4 $$\Delta {E}_{p}={E}_{pM}\left(1-\frac{{Z}_{i}}{{A}_{i }}\right),$$

And for $${\mathrm{A}}_{\mathrm{i }}\ge 2{\mathrm{Z}}_{\mathrm{i}}$$:

5 $$\Delta {E}_{p}\ge \frac{{E}_{pM}}{2},$$

So, for a given experiment of TNSA mechanism, or in general for any laser-matter process where ions are accelerated because of a potential drop, we can consider as a general criterion that if in a TOF scheme we are able to detect the maximum proton energy ${E}_{pM}$, then at least in the detected proton energy range $\left({E}_{pM}/2;{E}_{pM}\right)$ there will be no contribution coming from the superimposition of other ions. This energy interval was also highlighted in the spectrum reported in Figs. 2c and 5. It is worth to consider that this estimation was achieved under the rather conservative hypothesis of fully stripped light ions, which is a very limiting case, since in typical experiments ${z}_{i}<{Z}_{i}$ for the largest part of the ions.

### Estimation of laser-to-proton energy conversion

From the spectrum reported in Fig. 2c we consider here a rather rough estimation for the total number of generated protons ${N}_{\text{p,tot}}$, the associated total energy ${E}_{\text{p,tot}}$ and the ratio between the overall proton energy and the laser energy *R*_L–P_, under the assumption of emission limited to a unit of solid angle, and uniform within it. Of course, this has limited accuracy^2–4^, and a more realistic determination of these amounts would require the simultaneous use of several detectors at different angles, capable to characterize the angular variation of the emitted ion spectra, according to what reported in literature^46,47,58^. Anyway, this is out of the purpose of the present work, and we use these estimations only as useful indicative parameters of the interaction regime. According to these premises, the following parameters were extracted: ${N}_{\text{p,tot}}=9.9\times {10}^{12}$/sr, ${E}_{\text{p,tot}}=0.3$ J/sr and *R*_L–P_ = $12\mathrm{\%}$. These values are indeed far above those known from the literature^46,47,58^. This large overestimation can be easily explained by taking into account that other particles besides protons contributed to the detected signal. In particular, by applying those considerations to the same shot #38, under the conservative assumption of fully stripped light ions reaching the diamond, it results (t_pM_–Δt_p_, t_pM_) = (65.4 ns, 92.4 ns) or, in terms of proton energy, (E_pM_–ΔE_p_, E_pM_) = (1.077 MeV, 2.154 MeV). By limiting the estimation of ${N}_{\text{p,tot}}$, ${E}_{\text{p,tot}}$ and *R*_L–P_ to this interval, the value $5.9\times {10}^{10}/\mathrm{sr }, 0.01\mathrm{ J}/\mathrm{sr}$ and $0.4\mathrm{\%}$ are obtained, respectively. These are sensibly lower than those obtained in the previous evaluation and also in better agreement with those reported in literature for similar experimental conditions^46,47,58^.

The analysis of the data collected from those shots where an aluminium filter was used provided a further confirmation of the obtained results and of the developed theoretical evaluations. Taking as reference the de-embedded signal obtained for the shot #5 (E_L_ = 2.65 J, I_L_ = 1.8 × 10^19^ W/cm^2^), reported in Fig. 4a, it is possible to analyse the effect of the filter. In the white region on the left, particles reaching the detector do not feel the effect of the filter remarkably, which is indeed effectively acting on those arriving at later times, i.e. in the red region. In particular, the signal detected for t > 100 ns is given by two main contributions. The first is from the bremsstrahlung radiation from the particles interacting with the filter. The second is the contribution of the particles with energies close to the cutoff, which are still able to pass through the filter but with a large reduction of their velocity and of their number, as described by Eq. (15) in the “Methods” section. The spectrum obtained for protons in the white region of Fig. 4a is illustrated in Fig. 4b. For shot #5 we obtain, ${N}_{\text{p,tot}}=9.8\times {10}^{10}/\mathrm{sr},$ ${E}_{\text{p,tot}}=0.02\mathrm{ J}/\mathrm{sr}$ and *R*_L–P_ = $0.7\mathrm{\%}$, in very good agreement with the previous case without the filter, when the simultaneous particle detection is properly considered.

Even with the application of the filter, the obtained spectrum is not completely free from heavier ion contribution. The simultaneous detection of protons and ions is the reason for the evident rise at low energies of the computed spectra in Fig. 4. This is not related to the proton acceleration process itself but is due to the contribution to the spectrum of carbon ions precisely in that energy range, which cannot be separated from the one of the protons. According to the previous discussion, for all those energies lower than ${E}_{p,M}/2$ the signal could be given by the sum of ions and protons impinging on the diamond detector. For instance, using the relation ${E}_{C}={m}_{C}/{m}_{p}\cdot {E}_{p,M}/2$ it is possible to compute the equivalent energy that a carbon ion detected with the same delay as a certain proton would have. For the spectrum given in Fig. 5b we have ${E}_{p,M}/2 = 1.153$ MeV, which means that if the signal were generated by carbon ions, they would have an energy ${E}_{C}=13.8$ MeV. As already mentioned the cutoff energy of the $10$ μm aluminium filter for carbon ions is $11.5$ MeV, hence we have a window where carbon ions are able to pass through the filter and reach the diamond detector, namely from $11.5$ to $13.8$ MeV or, in terms of protons’ energy, from $0.953$ to $1.153$ MeV. Actually, as already discussed for protons, the carbon ion passing through the filter will be slowed down, resulting in an additional delay between different ion species. For instance, in the case under investigation we see that, using Eq. (16), the time needed for protons of $1.153$ MeV to reach the detector is $\simeq 9.1$ ns, while the time needed for carbon ions of equivalent energy is $\simeq 11.3$ ns, which means that they are going to affect the signal generated by protons of 0.906 meV. In other terms the spectral range affected by carbon ions results to be shifted from $(0.953$, $1.153$) MeV to (0.750, 0.906) MeV. Similar consideration should be applied for heavier ions.

## Discussion

In this work we reported on the details of an effective and accurate TOF measurements in a highly EMP-polluted environment. The described methodology allowed to optimize the experimental set up and the acquisition system in order to get a high signal-to-noise ratio on the whole dynamic range of the collected signals; this was achieved thanks to the high rejection to the EMP fields reached in the described way. The tailored optimization of the analogic signal management enabled the retrieving of both the interaction-time and the particle information from the recorded data with high accuracy.

The TOF method was here used to get information on the pure proton contribution from all the particle species detected by diamond detector. Measurements and data retrieving were performed promptly for every shot, without any need of opening the vacuum chamber. Detailed considerations were here presented for getting the calibrated proton spectrum associated to the time-domain measurements. The results of the theoretical discussion were applied to experimental data for shots performed with ~ 120 TW power and $2\times {10}^{19}$ W/cm^2^ intensity. A novel detailed procedure was applied for getting accurate spectra from the diamond detector, by taking into account its efficiency as a function of the energy of the impinging particle, obtained by the experimental calibrations of its response to protons of different energy.

Moreover, it was here shown that the use of tailored filters can positively decrease the lower value of proton spectrum, in general at some expenses of the tolerance. This can be anyway improved for filters where the thickness is known or determined with high accuracy. In the described experimental campaign, the advanced TOF method allowed to obtain calibrated spectra of protons up to ~ $2.5$ MeV with very high accuracy, much better than that typically obtained with Thomson spectrometers^38,59^. It is worth to notice that the approaches here followed for estimating the range where the detected signal is assumed to be purely due to protons—based in one case on established theoretical considerations^2–4,41^ and in the other case on the use of a suitable filter—results to be rather conservative, if compared with the energy spectra given in reference^60^. In that case data obtained by Thomson spectrometers, employed in experimental campaigns at very similar regimes to that described in the present paper, showed that the energy of carbon ions was significantly lower than the minimum proton energy considered in the present analysis^60^. Hence, for future development of the technique discussed here, its simultaneous employment together with a Thomson spectrometer, placed at similar detection angle, will be fruitful for extending the effective energy range where a pure proton spectrum is retrievable.

The capability of performing such on-line accurate measurements in these environments with high EMP levels but without the use of Faraday cages, is of crucial importance to monitor and characterize effectively the particles accelerated via laser-plasma interaction in high-repetition laser-matter experiments. This is a key point for high-intensity and high-energy laser facilities for both laser-plasma acceleration and inertial confinement fusion (PETAL, Vulcan Petawatt, Extreme Light Infrastructure (ELI)), where high levels of EMP are classically produced. Since the EMP fields are known to scale with laser energy and intensity^15^, the problem will be of increasing importance for future facilities which will be operating soon (Apollon 5 PW, ELI L4 10 PW, …). where higher EMP levels are expected. The technique was tested at FLAME laser and, in order to understand its possible application to facilities affected by even higher EMP pollution levels, some preliminary and successful tests were performed in experiments at the Vulcan Petawatt laser^61^ with laser energy ${E}_{L}=500$ J, pulse width 500 fs, ${I}_{L}\cong {10}^{21}$ W/cm^2^, and also very recently at the Phelix laser with ${E}_{L}=100$ J, pulse width 750 fs, ${I}_{L}\cong {10}^{20}$ W/cm^2^ (these latter results will be presented in a separate publication).

The potential high energy resolution is one of the key factors of the TOF technique and was here proved by using thin-fast diamonds in an advanced implementation of this scheme.

By using these advanced and optimized methodologies, fast diamond detectors can be thus successfully adopted as a main constituent of a layered diamond detector system made of a stack of them. Such configuration will allow to get a composite detector with effective thickness of extended dimension without losing energetic resolution and featured by high radiation hardness, thanks to the independent thin diamonds available throughout the structure. The single module was here studied and characterized in an energy range where protons were completely stopped inside the active diamond layer. This allowed to evaluate its temporal response leading to the high energy resolution and to retrieve the proton spectra exploiting the performed offline calibration. Protons with higher energies will leave only a portion of this energy to the diamond layer. As discussed in this work, for them a layered structure of active detectors is the key for a tailored advanced detection system, capable to reconstruct the proton spectrum with high energy resolution and with all the significant advantages discussed for this single diamond module, which will be extended to a wider range of particle energies.

These considerations confirm that the described methodology, together with the overall advanced associated detector system, is a promising candidate for the fast, accurate, high-resolution, high sensitivity, high radiation hardness and online detection of energetic ions in experiments of high energy and high intensity matter interaction with ultra-short laser pulses up to the femtosecond and even future attosecond range.

## Methods

### Single-module diamond

The detector was developed and optimized to work in the harsh environments of high-power and high-energy laser-matter interactions^29^, where high levels of EMP fields are generated. Thanks to their wideband gap of 5.5 eV, their high carrier mobility, and their radiation hardness^42,43,62,63^, diamonds are particularly useful to monitor UV, X photon emission and accelerated particles in laser-plasma experiments^29,43,64–66^. Indeed, they have low leakage current, fast time-response, and insensitivity to visible and infrared light which avoids any effect due to possible coupling of any stray light coming from the main laser beam to the detector. This is important to get an intrinsic low background level. To fully investigate the management of the EMP issue in the most demanding conditions, we used here a fast detector consisting of a 50 μm intrinsic diamond layer grown by chemical vapour deposition technique on a commercial 4 mm × 4 mm × 0.5 mm High Pressure High Temperature (HPHT) substrate. The detector operates in planar configuration with superficial interdigital aluminium contacts with 20 µm width and 20 μm spacing^29^, which allow for fast time detection of moderate energy particles. The time resolution of this detector was offline obtained from detection of single α particles of ${E}_{\mathrm{\alpha }}=5.486$ MeV energy, coming from ^241^Am source, and it resulted Δt = 0.8 ns^38^. By considering a Gaussian fitting of the associated single-particle detected signal, this means a 3-dB frequency bandwidth ${f}_{3dB-diamond}$ = 625 MHz.

The diamond detector was linked to the splitter through a cable connected with a custom large-capacity, high-frequency Millimetrica Bias-Tee (MIL-BT 010-6000). This isolated the scope from the − 150 V bias voltage for the diamond, without losing significant information over the frequency range (300 kHz–6 GHz), suitable and tailored for signals coming from the diamond detector.

### TOF for high dynamic-range detection

The splitting of the main diamond signal in two halves by a calibrated splitter, as shown in Fig. 2a, allowed to have a suitable characterization of its intrinsic high dynamic range. On this purpose, each of them was stored on a separate oscilloscope channel. The high dynamic-range LECROY HDO 4104 scope (1 GHz bandwidth, 10 Gs/s sample rate per channel, 12-bit resolution, 8.4 Effective Number Of Bits (ENOB)) was used for the measurements, with different amplitude scales for each of the two channels. The use of a high-resolution and high-sensitivity oscilloscope such as the HDO4104 is important for the improvement of the dynamic-range of this type of acquisitions. The intrinsic dynamic range of the used low-noise oscilloscope is indeed already high, but with this technique it was possible to improve it of a factor ~ 1.4. This configuration allowed to obtain an actual dynamic range of ~ 74 dB. Of course, the overall dynamic range obtained with this technique can be higher when oscilloscopes with lower intrinsic noise are used.

### Calibrated spectrum determination: from data acquisition to spectrum reconstruction

The determination of accurate energy spectra for incoming particles from the TOF signals generated by the detector is a delicate issue. In some cases, simple mathematical reasoning is used to get the energy-domain spectra from the stored time-domain signals, as very recently reported^﻿﻿24^. Anyway, they do not consider the specific experimental response of the detector to a specific particle with a specific energy.

We describe here instead a detailed procedure for getting accurate spectra from a diamond detector, including the experimental calibrations of its response. First, in the next section, a detailed description of the EMP management, allowing to obtain signals almost free from electromagnetic noise, is reported. Then we show the procedure used to include in the results the effects due to the long cables employed to manage the EMP pollution affecting the diamond signal. Eventually, a novel methodology is described to get the calibrated energy spectrum. To this purpose, we analyze the response of the diamond detector and discuss in detail also the overall spectrum accuracy for the energy and the number of particles.

### EMP management and reduction

**Figure 6:**
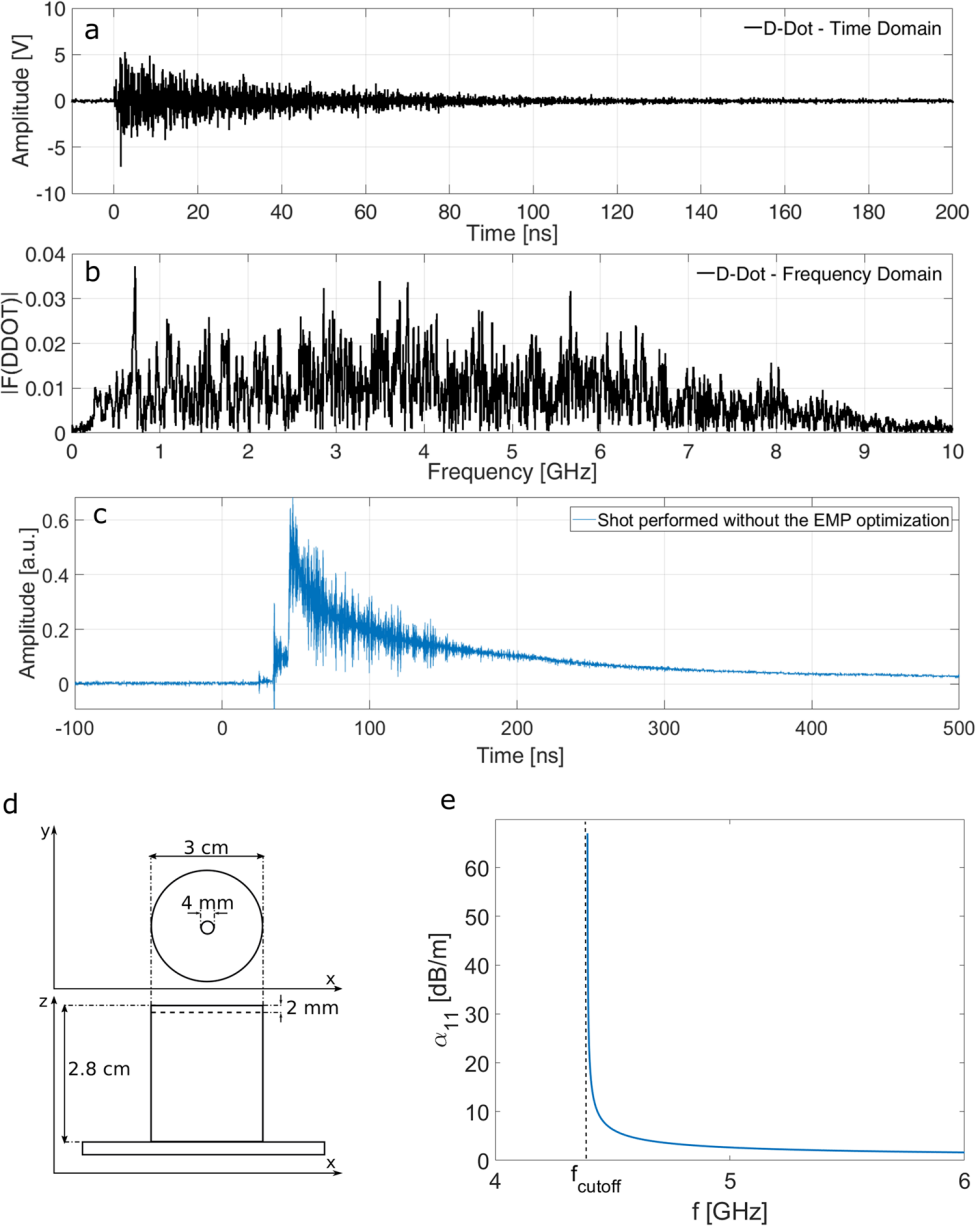
(**a**, **b**) EMP measurement obtained by the D-Dot probe: (**a**) time domain (**b**) frequency domain obtained by means of Fourier Transform. (**c**) An example of the time domain signal collected without the discussed optimization for the EMP and performed in the same experimental condition as the signal shown in Fig. 2b. (**d**) Scheme of the EMP-optimized case of the diamond detector. (**e**) Attenuation coefficient α for the TE_11_ mode provided by a cylindrical pipe as a function of the frequency, with indication of the cutoff frequency.

It is well known that in high power laser-matter interactions the effect of the EMPs on diagnostics with electronical components can be remarkable^12–18^. This generally depends on the specific type of laser matter interaction and its intensity is known to scale with the laser energy and intensity on focus^12,41^. There are several mechanisms of generation for the intense EMP fields in the experimental conditions of this campaign, with a classical TNSA scheme^12,67^. The common main source is the neutralization current flowing through the target holder to the chamber floor, caused by the fast electron emission from the target due to the intense laser-matter interaction. A typical EMP signal, measured inside the experimental vacuum chamber during the present experimental campaign, is shown in Fig. 6a. It was obtained in a shot at maximum laser energy and intensity on an Al foil target, and detected by a customized version of the AD-80D(R) D-Dot differential electric field sensor^14,32^ (3-dB-bandwidth up to 5.5 GHz). It was positioned at 31 cm from the target and had 15 cm height from the chamber floor and -3 cm shift in the *x* direction with respect to the target position, as shown in Fig. 1c. The sensing direction of the probe was radially oriented to the target, and a 5 cm thick lead block was used to shield the D-Dot from ionizing radiation generated by the interaction. The BIB-100G balun (250 kHz–10 GHz bandwidth) was connected to its terminals. The Tektronix DPO72004B (16 GHz, 50 GS/s) fast oscilloscope was used to store the acquired data. In Fig. 6b the frequency spectrum of the detected signal, obtained by suitable Fourier Transform, is also given. It is apparent that the produced EMP had a very wide band. In particular, it exceeded the 3 dB bandwidth of the D-Dot probe. So, for the frequency components higher than 5.5 GHz a single-pole attenuation has to be considered^12^ (a paper is in preparation about this issue). By following the procedure described in detail in reference^17^, from the acquired measurement it was possible to get an estimation of the associated maximum peak-to-peak electric field ~ 25 kV/m, that is coherent with what expected for these experimental conditions^12,41^. Such high level of EMP fields produced, in the first test shots of the campaign, diamond signals strongly affected, and sometimes even hidden, by the EMP oscillations^68^. One of the diamond signals collected during these first shots is shown in Fig. 6c. The laser-matter interaction in this shot had the same typical parameters of those reported in Figs. 2 and 4, but the detection system was not yet optimized to work in presence of strong EMPs.

Several precautions were here adopted to obtain signals from the diamond detectors with high signal-to-noise ratio even in these environments with high electromagnetic pulse pollution. In particular, two types of EMP field coupling had to be avoided: that directly with the detector and that with the overall acquisition system. We give here details on how the implemented techniques acted on the EMP fields and their coupling to the whole diamond-detector measurement setup.

The EMP coupling with the device was minimized thanks to an ad-hoc mounting structure^29^ of the diamond detector holder shown in Fig. 6d. The diamond was mounted inside a compact cylindrical metallic enclosure with a minimal circular aperture on the front. The small radius *r* provides a cutoff frequency, ${\mathrm{f}}_{\mathrm{cutoff}}\propto \mathrm{c}/\mathrm{r}$, where *c* is the speed of light, which highly limits the EMP coupling without covering the active surface of the diamond detector^69^. The same principle was applied to the TOF line. The final connection with the diamond detector is a pipe of 65 cm length and ${\mathrm{R}}_{\mathrm{TOF}}=20$ mm internal radius, as shown in the main picture of Fig. 1. This acted as a cylindrical waveguide, and in particular for the first waveguide mode TE_11_ the cutoff frequency was: ${\mathrm{f}}_{\mathrm{cutoff}}={\mathrm{c}}{\dot{\upchi }}_{\mathrm{1,1}}/\left(2\uppi {\mathrm{R}}_{\mathrm{TOF}}\right)=4.395$ GHz, where ${\dot{\upchi }}_{\mathrm{1,1}}= 1.841$ is the first root of the derivative of the first Bessel Function. The field intensity associated with a given mode decreases along the waveguide longitudinal direction *z* with dependence ${\mathrm{e}}^{-\mathrm{\alpha z}}$. The attenuation coefficient α for the TE_11_ mode as a function of the frequency is shown in Fig. 6e. A very good rejection to EMP fields traveling in the vacuum chamber was thus achieved.

**Figure 7:**
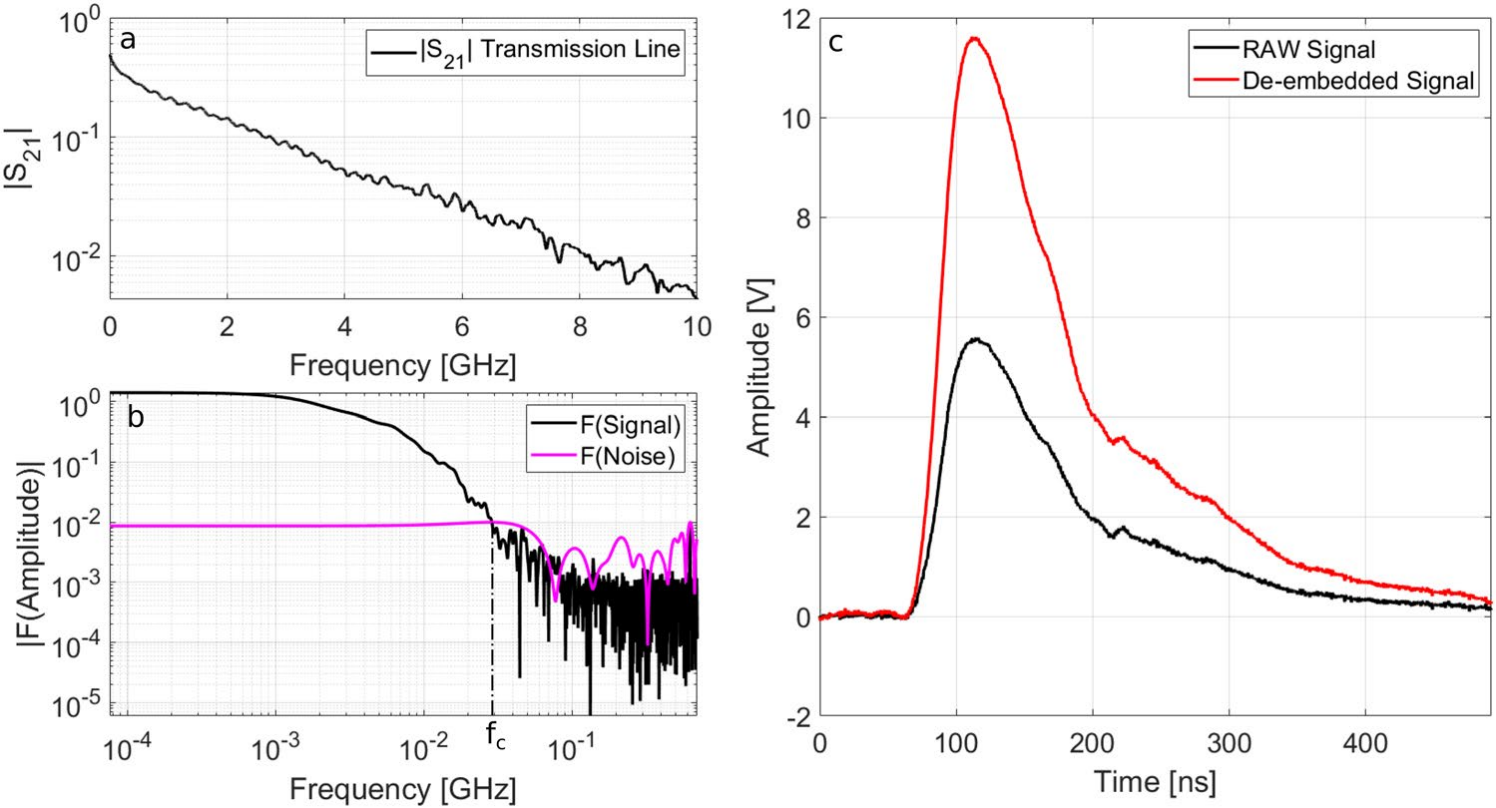
(**a**) The Scattering parameter S_21_ of the transmission line (coaxial cables, splitter and bias tee) measured with the Agilent N5230A Network Analyzer (**b**) Fourier transform of the acquired signal and of the background noise (**c**) De-embedded signal ${\mathrm{S}}_{\mathrm{D}}$ compared with the original raw V(t), equal to the double of the signal detected on Ch3.

In order to reduce the EMP coupling with the acquisition system, the main transmission link consisted of ~ 15 m RG223 double-shielded coaxial cables, used to transport the signal from the detector to the scope. They have a shielding effectiveness higher than 80 dB for the frequency ranges related to the performed experiments and resulted very suitable to suppress the direct transmission of the EMP fields traveling in air to the inner coaxial conductor of the cable, through their outer shielding conductor. The coaxial cables, together with the calibrated splitter and the custom bias-tee, were offline characterized in the frequency domain by the Agilent N5230A Vector Network analyser. The obtained S_21_ scattering parameter is given in Fig. 7a.

These long cables worked on the EMP rejection in several ways.

- They behaved as effective low-pass filters^16^, giving high attenuation to possible contributions at high frequencies. On one side this improved the filtering of the high-frequency components of possible EMPs coupled to the detector. But on the other side it might have introduced limitations to the fast response of the detector setup. As observed, the 3 dB low-pass bandwidth of the interdigital diamond detector resulted 625 MHz. According to Fig. 7a, the S_21_ scattering parameter for this frequency was 0.2492 (~ 12 dB), which indeed resulted a very suitable compromise.
- The propagation of the EMP fields outside the experimental chamber can be classically modelled with the 1/r law. The use of long cables allowed the positioning of the scopes far from the experimental chamber, and thus decreased the possible direct coupling of EMP fields with the scopes themselves^12,16^.
- EMPs have a typical exponentially-decreasing time profile and become usually comparable to the background noise in ~ 100 ns, as shown in Fig. 6a. Since signals traveling in cables have lower velocity than those traveling in air, the cables were also used to introduce a temporal delay of several tens of nanoseconds between the high EMP contribution and the detected signal^12,16^.

Those cables were also surrounded by several ferrite toroids, with bandwidths 0.5–5 MHz, 1 MHz–1 GHz, 2–30 MHz, 20–200 MHz, respectively, providing damping of the current flowing on the outer conductor of the cables caused by the applied external EMP fields. This avoided that those currents could reach the oscilloscopes and generate EMP noise coupled directly to it.

By means of these methods it was possible to obtain detected diamond signals with high signal-to-noise ratio. The EMP high frequency oscillations shown in Fig. 6a, with spectrum represented in Fig. 6b, are practically absent in the diamond signals shown in Figs. 2, 4 and 5, and this was achieved without any numerical filtering procedure and without the use of expensive high-quality Faraday cages.

### Cable de-embedding procedure

The signal collected by the CVD diamond detector was transmitted to the scope through the long coaxial link, having a frequency-dependent attenuation. Thus, a suitable de-embedding procedure^17^ was required to recover the signal at the detector site from the one recorded on the oscilloscope. The de-embedded signal ${S}_{D}$ can be computed by the following relation^17^:

6 $${S}_{D}\left(t\right)={\mathcal{F}}^{-1}\left[\frac{\mathcal{F}\left(V\left(t\right)\right)}{{S}_{21}\left(f \right)}\right]$$

where $V(t)$ is the signal stored on the scope and $\mathcal{F}$ and ${\mathcal{F}}^{-1}$ are the Fourier transform and the inverse Fourier transform operators, respectively. As a preliminary step, the Fourier transform of the signal and that of the background noise were computed and compared (see Fig. 7b) to determine the bandwidth where a suitable signal-to-noise ratio was actually achieved. With the assumption of white-noise condition, the information about the background noise was retrieved from the portion of the signal collected in the time interval before the photopeak detection instant, when the laser-matter interaction is not occurred yet. Then, the frequency where the amplitude of the Fourier transform of the noise was equal to the Fourier transform of the signal, was selected as threshold frequency ${f}_{c}$ (highlighted in Fig. 7b) and used to identify the frequency range in which the de-embedding procedure could be suitably applied. This preliminary step was necessary to avoid numerical amplification of the noise, and therefore Eq. (6) was applied only to the meaningful part of the acquired signal.

In Fig. 7c the final de-embedded signal ${S}_{D}$ is obtained for the shot #38 and compared with the original raw signal V(t); it is evident that the contribution of the transmission line cannot be ignored.

### Analytical spectrum computation

#### Particle energy estimation

To retrieve the accelerated particles spectrum, as a first step it is necessary to define an absolute time-reference related to the laser-matter interaction instant. As discussed, at the moment of laser interaction with matter, a burst of UV-X rays is generated and detected by the diamond detector. The detection time ${\mathrm{t}}_{\mathrm{ph}}$ of this narrow photopeak, shown in Fig. 2b for shot #38, can be thus used as absolute time reference. The time needed for photons to travel from the source to the detector is $\Delta {\mathrm{t}}_{\mathrm{prop}}=\mathrm{d}/\mathrm{c}$, being *d* the distance of the detector from the interaction point. Therefore, the time instant when the laser-matter interaction occurs can be determined as ${\mathrm{t}}_{\mathrm{bang}}={\mathrm{t}}_{\mathrm{ph}}-\Delta {\mathrm{t}}_{\mathrm{prop}}$, and used as reliable absolute reference. We indicate with ${\mathrm{t}}_{\mathrm{i}}$ the ion detection time according to the new temporal reference and with v_i_ = d/t_i_ the associated detected flight velocity. In this way, by TOF methods accurate information on particle velocity can be achieved. From these measurements calibrated particle spectra can be obtained, if the type of particle reaching the detector is known or assumed for a given time interval^38^. Under this condition, for a generic ion species the energy associated with the detection time can be obtained from the relation: ${\mathrm{E}}_{\mathrm{i}}=\left({\upgamma }_{\mathrm{i}}-1\right){\mathrm{m}}_{\mathrm{i}}{\mathrm{c}}^{2}$, where ${\upgamma }_{\mathrm{i}}$ and ${\mathrm{m}}_{\mathrm{i}}$ are the ion associated relativistic factor and its mass, respectively.

The finite temporal resolution Δt of the detector, obtained with the mentioned calibration, affects the energy tolerance of the measurement performed with the diamond detectors. For a generic time interval, indicated with the *n* index, with ${t}_{n}^{i}$ and ${t}_{n}^{f}$ initial and final extremes, will be $\Delta t={t}_{i,n}^{f}-{t}_{i,n}^{i}$ and ${E}_{i,n}^{i}$ and ${E}_{i,n}^{f}$ the associated ion energies. Thus, the error on the energy estimation will be given by:

7 $$\Delta {E}_{i,n}={E}_{i,n}^{f}-{E}_{i,n}^{i}=\frac{\partial {E}_{i}}{\partial \gamma }\Delta {\gamma }_{i}={m}_{i}{c}^{2}\frac{\partial {\gamma }_{i}}{\partial t} \Delta t$$

The relative error on energy, given by the finite temporal resolution, can be written as:

8 $$\frac{\Delta {E}_{i,n}}{{\stackrel{-}{E}}_{i,n}}=-\frac{{m}_{i}{c}^{2}}{{\stackrel{-}{E}}_{i,n}}\left(\frac{c\Delta t}{d}\right){\left[{\left(\frac{{\stackrel{-}{E}}_{i,n}}{{m}_{i}{c}^{2}}+1\right)}^{2}-1\right]}^{3/2}$$

where ${\stackrel{-}{\mathrm{E}}}_{\mathrm{i},\mathrm{n}}= \sqrt{{\mathrm{E}}_{\mathrm{i},\mathrm{n}}^{\mathrm{f}}{\mathrm{E}}_{\mathrm{i},\mathrm{n}}^{\mathrm{i}}}$ is the average ion energy in the *n*^*th*^ interval. The negative sign is because energy is a monotonically decreasing function of time. In case of non-relativistic particles ${\stackrel{-}{\mathrm{E}}}_{\mathrm{i},\mathrm{n}}\ll {\mathrm{m}}_{\mathrm{i}}{\mathrm{c}}^{2}$, and the previous relation can be simplified obtaining:

9 $$\frac{\Delta {E}_{i,n}}{{\stackrel{-}{E}}_{i,n}}=-2\sqrt{2}\frac{\Delta t}{d}\sqrt{\frac{{\stackrel{-}{E}}_{i,n}}{{m}_{i}}}=-2\frac{\Delta t}{{\stackrel{-}{t}}_{i,n}}$$

where ${\stackrel{-}{\mathrm{t}}}_{\mathrm{i},\mathrm{n}}= \sqrt{{\mathrm{t}}_{\mathrm{i},\mathrm{n}}^{\mathrm{f}}{\mathrm{t}}_{\mathrm{i},\mathrm{n}}^{\mathrm{i}}}$ is the average time in the *n*th interval. Thus, the absolute value of the relative error on the energy is equal to twice the relative error on the measurement of the time of ion arrival to the detector. So, for the same $\Delta t$ mainly due to the time resolution of the detector, the accuracy will be better for particles arriving to the detector at later times, so for detectors placed at larger distances from the target. But this, on the other hand, would decrease the number of particles hitting the same detector, and thus the signal amplitude.

#### Determination of the particle number

As already mentioned, the interdigital diamond configuration consists of a 50 μm intrinsic diamond layer, capable to fully stop protons with energy up to ~ 3 MeV. The signal generated by incoming protons is proportional to the particle energy. The induced charge signals collected at the sensitive electrode can be considered as due to the motion of electron/hole pairs generated in the bulk of the diamond sample, and charge pulses are formed only in regions where an electric field (E) is present. For this reason, the region where the charges are more efficiently collected is that closer to the electrode surface. As a consequence, the response of the interdigital diamond detector is high for particles with energies low enough to be completely stopped inside this high-efficiency layer, and it starts to decrease for those having higher energies, and thus larger ranges, up to the case where they will generate a signal on the detector comparable to the noise level. Indeed, it is well known that the charge collected in diamond detectors due to ${N}_{i}$ incident particles, depositing energy ${E}_{i}$ in the sensitive layer, can be expressed by ${Q}_{i} ={\eta }_{i}\left({E}_{i}\right){q}_{e}{N}_{i}{E}_{i}/\epsilon$, where ${q}_{e}$ is the electron charge, $\epsilon$ is the average electron–hole pair energy creation, i.e. 13 eV for the diamond, and ${\eta }_{i}\le 1$ the Charge Collection Efficiency (CCE) of the detector for a given particle^62^.

In order to evaluate the CCE curve, the diamond detector was previously characterized at the AN2000 microbeam facility of the National Laboratories of Legnaro (Italy) by using proton beams of energy respectively 0.3 MeV, 1 MeV, 1.4 MeV and 2 MeV. The beam was focused to a spot size of ~ 5 μm on the sensitive electrodes of diamond. As evaluated by the SRIM Monte Carlo Simulation code^39^, the range of protons in diamond varies from 1.5 to 25 μm. An electronic chain consisting of a charge-sensitive preamplifier ORTEC142A and an ORTEC572 shaping amplifier was used. The calibration of the electronic chain was performed using a Si detector and a precision pulse generator ORTEC419 relating the pulse heights provided by the reference Si detector with those from the diamond devices.

**Figure 8:**
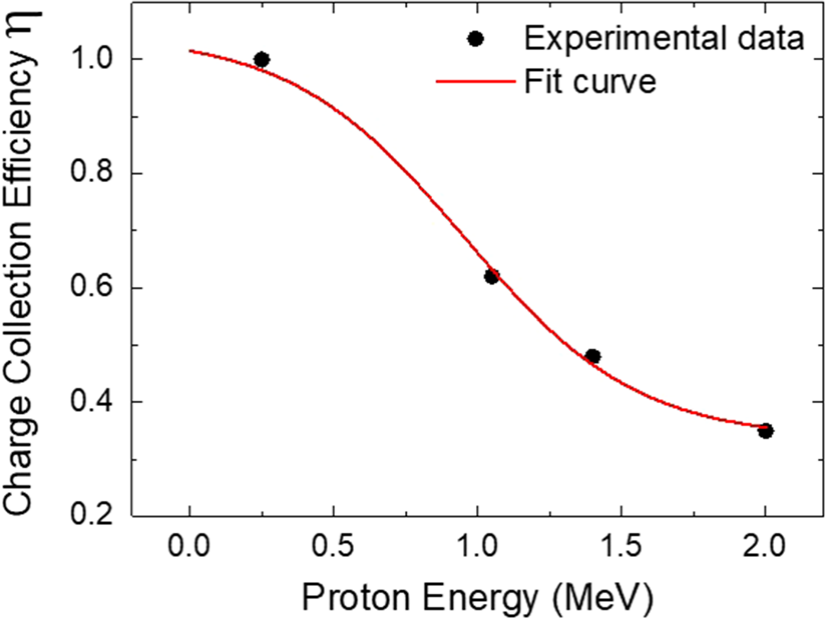
Charge collection efficiency (CCE) of the diamond detector as a function of energy. Fit curve is also reported in the figure.

The CCE of the detector as a function of the investigated proton energies is reported in Fig. 8.

As expected, for low energy protons, the induced charge is equal to the generation charge (i.e. $\eta =1$) whereas, for high energy protons, charges are generated in the region where weak electric field is present, and therefore the CCE of the detector decreases. The probability for free carriers to enter the depletion region exponentially decreases, with a logarithmic slope equal to their diffusion length. The experimental data are well fitted by a non-linear curve having the following expression:

10 $${\eta }_{i}\left({E}_{i}\right)=\frac{0.71}{1+{e}^{\frac{{E}_{i}-9.1{\times 10}^{5}}{3.1{\times 10}^{5}}}}+0.33$$

By considering that equal CCE is obtained for other particles having the same range in the detector, from these measurements it is possible to determine the CCE also for other ion species.

The number of incoming ions of known specie can be retrieved according to the following relation:

11 $${\mathrm{N}}_{\mathrm{i}}=\frac{{Q}_{i} \epsilon }{\varphi \left({E}_{i}\right){ q}_{e}}$$

where the definition $\varphi \left({E}_{i}\right)\equiv {\eta }_{i}\left({E}_{i}\right) {E}_{i}$ is used for simplicity. In a generic *n*th time interval, the value of the associated charge generated in diamond can be computed:

12 $${\stackrel{-}{Q}}_{i,n}=\frac{1}{R}{\int }_{{t}_{i,n}^{i}}^{{t}_{i,n}^{f}}{S}_{D}\left(t\right)dt$$

where *R* is the characteristic load impedance and ${\mathrm{S}}_{\mathrm{D}}\left(t\right)$ is the de-embedded signal. Also for the number of incoming ions it is possible to determine the uncertainty in the determination of ${\mathrm{N}}_{\mathrm{i}}$. In particular, Eq. (11) can be modified as follows:

13 $${\stackrel{-}{N}}_{i,n}=\frac{{\stackrel{-}{Q}}_{i,n} \epsilon }{{\stackrel{-}{\varphi }}_{i,n}{ q}_{e}}$$

where ${\stackrel{-}{\mathrm{N}}}_{\mathrm{i},\mathrm{n}}$ and ${\stackrel{-}{\varphi }}_{i,n}$ are the average of the number of ions intercepting the detector in the *n*th time interval, and the related average of the $\varphi$ function in the same interval. As a consequence, the relative error on the number of incoming ions, given by the finite temporal resolution, can be written as:

14 $$\frac{\Delta {N}_{i,n}}{{N}_{i,n}}=\frac{{N}_{i,n}\left({E}_{i,n}^{f}\right)-{N}_{i,n}\left({E}_{i,n}^{i}\right)}{{\stackrel{-}{N}}_{i,n}}={\stackrel{-}{\varphi }}_{i,n}\left[\frac{1}{\varphi \left({E}_{i,n}^{f}\right)}-\frac{1}{\varphi \left({E}_{i,n}^{i}\right)}\right]$$

Hence, the error on the estimation of the number of impinging particles can be expressed in terms of the $\varphi$ function and is thus strongly related to the temporal resolution of the detector. Nevertheless, it is important to underline that the performed evaluation holds on the assumption that only one kind of particle is entering the detector.

#### Filter application

In general, to retrieve the proton spectrum in case a filter is used between the target and the detector, it is necessary to correct the number of particles estimated from Eq. (11) as it follows:

15 $${\mathrm{N}}_{\mathrm{i}}=\frac{{Q}_{i} \epsilon }{{k}_{att}\left({E}_{i}\right) \varphi \left({E}_{i}\right){ q}_{e}}$$

and also the related Eqs. (12) and (13) accordingly, where the attenuation factor ${k}_{att}\left({E}_{i}\right)$ was here retrieved by SRIM simulations. Moreover, in a classical TOF scheme, the particle energy is easily linked to a specific time instant from the equation of uniform rectilinear motion. When a filter is used, the overall path is schematically split in two consecutive ones, where the same particle has different velocities. The first, having length D, from the target to the filter, and the second of length $\updelta$, from the filter to the detector. In general, the overall arrival time ${\stackrel{-}{\mathrm{t}}}_{\mathrm{p},\mathrm{n}}$ of a proton to the detector can be thus written as:

16 $${\stackrel{-}{t}}_{p,n}={\stackrel{-}{t}}_{pD,n}+{\stackrel{-}{t}}_{p\delta ,n}=D\sqrt{\frac{{m}_{p}}{2{\stackrel{-}{E}}_{p,n}}}+\delta \sqrt{\frac{{m}_{p}}{2{k}_{att}\left({\stackrel{-}{E}}_{p,n}\right){\stackrel{-}{E}}_{p,n}}}=\sqrt{\frac{{m}_{p}}{2{\stackrel{-}{E}}_{p,n}}}\left(D+\frac{\delta }{\sqrt{{k}_{att}\left({\stackrel{-}{E}}_{p,n}\right)}}\right)$$

This relation actually links arrival time and energy in the case a filter is used, and can be inverted numerically to find the associate ${\stackrel{-}{E}}_{p,n}$, since ${k}_{att}$ is a monotonic function to be obtained by offline experimental calibration of the filter or numerical simulations. It is clear that the second term will be of interest only for particles with energy close to the filter cutoff.

As a rule of thumb we can consider that the effect of the drift space located after the filter starts to be relevant when the time needed by a particle to travel through it is one tenth of the time needed to travel the main drift space D. To evaluate for which proton energy this happens it is convenient to express the filter attenuation factor as a function of the parameter $G={\stackrel{-}{t}}_{p\delta ,n}/{\stackrel{-}{t}}_{pD,n}$:

17 $${k}_{att}\left({\stackrel{-}{E}}_{p,n}\right)={\left(\frac{\delta }{DG}\right)}^{2}$$

In the present experiment $\delta =0.2$ cm and *D* = 105 cm; a value of $G = 0.1$ was thus achieved for ${k}_{att} = 3.64\times {10}^{-4}$. This value of the attenuation factor, as shown in Fig. 3a, is obtained for those protons with energies near the filter cutoff or lower than that.

We can conclude that in the present study the effects of the drift $\delta$ can be neglected, but it is important to take into account the energy attenuation in Eq. (15). Nevertheless, Eq. (16) is very useful for all those cases where the filter is placed not too close to the detector, or when it is important to have rather accurate ion energy estimations very close to the filter cutoff.
